# Baseline α-synuclein seeding activity and disease progression in sporadic and genetic Parkinson's disease in the PPMI cohort

**DOI:** 10.1016/j.ebiom.2025.105866

**Published:** 2025-08-06

**Authors:** Jackson G. Schumacher, Xinyuan Zhang, Eric A. Macklin, Jian Wang, Armin Bayati, Johannes M. Dijkstra, Hirohisa Watanabe, Michael A. Schwarzschild, Marianna Cortese, Xuehong Zhang, Xiqun Chen

**Affiliations:** a Department of Neurology, Massachusetts General Hospital and Harvard Medical School, Boston, MA, 02129, USA; b Aligning Science Across Parkinson's (ASAP) Collaborative Research Network, Chevy Chase, MD, 20815, USA; c Department of Psychology and Neuroscience, Morrissey College of Arts and Sciences, Boston College, Chestnut Hill, MA, 02467, USA; d Channing Division of Network Medicine, Brigham and Women's Hospital and Harvard Medical School, Boston, MA, 02115, USA; e Massachusetts General Hospital Biostatistics, Massachusetts General Hospital, Somerville, MA, 02145, USA; f Department of Biostatistics, The University of Texas MD Anderson Cancer Center, Houston, TX, 77030, USA; g Department of Neurology, School of Medicine, Fujita Health University, Toyoake, Japan; h Department of Epidemiology, Harvard T.H. Chan School of Public Health, Boston, MA, 02115, USA; i Yale University School of Nursing, Orange, CT, 06477, USA; j Department of Nutrition, Harvard T.H. Chan School of Public Health, Boston, MA, 02115, USA

**Keywords:** Parkinson's disease, α-synuclein seed amplification assay, Progression, LRRK2, GBA

## Abstract

**Background:**

α-Synuclein (α-syn) seed amplification assays (SAAs) have shown remarkable potential in diagnosing Parkinson's disease (PD). Using data from the Parkinson's Progression Markers Initiative (PPMI) cohort, we aimed to test whether baseline α-syn seeding activity and α-syn SAA kinetic parameters are associated with disease progression in sporadic PD, *LRRK2*-associated PD (*LRRK2* PD), and *GBA*-associated PD (*GBA* PD).

**Methods:**

We analysed 7 years of motor, non-motor, and cognitive assessments and 5 years of dopamine transporter imaging along with baseline α-syn SAA results from 564 PPMI participants (n = 332 sporadic PD, 162 *LRRK2* PD, and 70 *GBA* PD) using linear mixed-effects models, adjusted for potential confounders, to test whether baseline α-syn SAA positivity (n = 315 sporadic PD, 111 *LRRK2* PD, and 66 *GBA* PD) and α-syn SAA kinetic parameters are associated with PD progression.

**Findings:**

While non-statistically significant, there was a trend towards faster motor decline in participants with α-syn SAA positive *LRRK2* PD compared to those with α-syn SAA negative *LRRK2* PD (MDS-UPDRS III points per year: 2.39 (95% confidence interval: 1.86–2.92) vs. 1.76 (0.93–2.60); difference = 0.63 (−0.29 to 1.55, p = 0.18). This trend appeared to be driven by R1441C/G + M1646T carriers (3.89 (1.22–6.55) vs. 0.31 (−1.32 to 1.93); difference = 3.58 (0.56–6.60, p = 0.02) and excluding them eliminated any trend (2.33 (1.79–2.86) vs. 2.26 (1.34–3.18); difference = 0.07 (−0.93 to 1.07, p = 0.89). Based on a clinically meaningful difference of 4.63 points we found no statistically significant or clinically meaningful difference in motor decline between α-syn SAA positive and α-syn SAA negative participants with sporadic PD (2.46 (2.20–2.72) vs. 2.39 (1.36–3.42); difference = 0.07 (−0.99 to 1.12), p = 0.90) or *GBA* PD (2.67 (1.91–3.44) vs. 2.40 (−0.18 to 4.99); difference = 0.27 (−2.42 to 2.96), p = 0.84). No statistically significant or clinically meaningful differences were seen in the progression of non-motor symptoms, cognition, or DAT imaging. Additionally, we found no clinically meaningful association between α-syn SAA kinetic parameters and PD progression.

**Interpretation:**

We found no statistically significant associations between baseline α-syn seeding activity, α-syn SAA kinetic parameters, and PD progression among manifest patients in the PPMI cohort. Future studies are needed to further investigate relationships among α-syn seeding activity, disease heterogeneity, disease stage, and PD progression.

**Funding:**

This research was funded by 10.13039/100018231Aligning Science Across Parkinson's grant ASAP-237603 through the 10.13039/100000864Michael J. Fox Foundation for Parkinson's Research and by the 10.13039/100000002National Institutes of Health through the 10.13039/100000065National Institute of Neurological Disorders and Stroke grants R01NS102735 and 5R01NS126260.

**Research in context**

**Evidence before this study**

We searched PubMed with the terms “Parkinson's disease,” “GBA,” “LRRK2,” “α-synuclein,” “seed amplification assay,” “seeding activity,” and “progression” for articles published on or before June 12, 2025. Many related articles were identified, particularly from the past four years, coinciding with the development of α-syn SAAs. However, few studies have investigated a direct association between α-syn seeding activity, α-syn SAA kinetic parameters, and PD progression.

**Added value of this study**

With well-characterised longitudinal clinical assessments and dopamine transporter imaging data, we report that baseline α-syn seed amplification assay (SAA) positivity and α-syn SAA kinetic parameters were not significantly associated with worsening motor, non-motor, or cognitive function or loss of dopamine transporter in sporadic and genetic forms of PD in the PPMI cohort.

**Implications of all the available evidence**

While α-syn SAAs have demonstrated utility as a research and diagnostic tool, whether they predict PD progression should be further investigated. Future studies, including earlier disease stages and longitudinal SAA changes, are needed to determine whether a relationship exists between α-syn seeding activity and PD progression.

## Introduction

Parkinson's disease (PD) is a progressive neurodegenerative disorder characterised by motor symptoms such as bradykinesia, resting tremors, stiffness, and impaired balance and coordination, along with non-motor symptoms, including cognitive decline, mood changes, and autonomic dysfunction.^1^ Pathologically, PD is primarily characterised by abnormally aggregated α-synuclein (α-syn) and degeneration of dopaminergic neurons in the substantia nigra (SN). A substantial body of evidence indicates that the aggregation of monomeric α-syn into pathogenic oligomeric confirmations and fibrils contributes to dopaminergic neurodegeneration and broader pathological processes in PD.^2^ Once these pathogenic aggregates form, they have been shown to propagate between cells, amplifying and accelerating disease pathology. The propagation of pathogenic α-syn aggregates is currently the prevailing theory behind the progressive nature of PD.^2^, ^3^, ^4^, ^5^, ^6^

Techniques to detect pathogenic α-syn seeds, collectively known as α-syn seed amplification assays (SAAs), leverage the ability of α-syn seeds to induce aggregation of recombinant α-synuclein monomers to enable their detection even at extremely low concentrations.^7^ α-Syn SAAs have shown high sensitivity and specificity when differentiating between sporadic PD and healthy controls.^7^ A recent study on phenoconversion in the Parkinson's Progression Markers Initiative (PPMI) found that α-syn SAA kinetic parameters, including time to 50% threshold (T50), time to threshold (TTT), and area under the curve (AUC) are faster and higher in patients with prodromal PD and manifest PD than in healthy controls.^8^ Additionally, multiple cross-sectional studies have shown that α-syn seeding activity is associated with more severe motor dysfunction, cognitive impairment, brain atrophy, and reduced connectivity in PD, as well as worse clinical outcomes in Alzheimer's disease (AD).^9^, ^10^, ^11^, ^12^ As such, α-syn SAAs have had a remarkable impact on the field and have prompted calls for a biological definition and staging system of PD.^13^^,^^14^ However, whether α-syn SAAs may provide information on PD progression remains largely unknown.

While the majority of PD cases are sporadic, variants in leucine-rich repeat kinase 2 (*LRRK2*) and glucocerebrosidase (*GBA*) are among the most common genetic risk factors for the disease.^15^^,^^16^ Previous studies have shown that pathogenic *LRRK2* gain-of-function variants are associated with slower PD progression, while *GBA* loss-of-function variants are associated with faster PD progression.^17^, ^18^, ^19^, ^20^ Interestingly, a study by PPMI reported significantly lower rates of α-syn SAA positivity in carriers of the *LRRK2* G2019S variant than those with sporadic PD.^21^ Here, we analysed clinical assessments and imaging data from 564 PPMI participants with baseline α-syn SAA results to determine whether α-syn seeding activity and α-syn SAA kinetic parameters are associated with progression in sporadic, *LRRK2*, and *GBA* PD.

## Methods

### Ethics

Ethical approval was not required as only publicly available data were used in this study.

### Analysis design and participants

This cohort analysis is based on data collected by the PPMI study and includes participants from the Parkinson's Disease Cohort and Genetic Registry. We did not include participants with prodromal PD as α-syn SAA data for those participants is not yet publicly available. We excluded participants who met enrolment criteria but had scans without evidence of dopamine deficiency (SWEDD) (n = 66). Genome-wide sequencing data from PPMI was used to categorize these participants into three groups: participants with sporadic PD (non-carriers of any known pathogenic mutations), participants with *LRRK2* PD (carriers of *LRRK2* G2019S, R1441C/G + M1646T (except for one, all R1441C/G carriers also carried an M1646T mutation and as such only those dual carriers were included), and N2081D/N14D variants), and participants with *GBA* PD (carriers of *GBA* E326K, R502C, A495P, and N409S variants). We excluded participants with *SNCA* (n = 38), *PRKN* (n = 9), *PINK1* (n = 1), *PARK7* (n = 1), and *VPS35* (n = 1) mutations, as well as those with both *LRRK2* and *GBA* mutations (n = 24).

The data analysed was collected by PPMI between the study's inception, July 7, 2010, and May 24, 2024. More detailed information about PPMI's mission and inclusion criteria, as well as sources and methods of participant selection, can be found on the PPMI website (www.ppmi-info.org/access-data-specimens/download-data). The data were last accessed using the PPMI portal on June 24, 2024.

### Clinical and SAA data

Participants underwent clinical assessments and imaging by PPMI-associated clinicians at baseline and during subsequent follow-up visits as previously described.^22^^,^^23^ Baseline refers to when participants were recruited into the cohort. Although PPMI has up to 14 years of Movement Disorder Society Unified Parkinson's Disease Rating Scale Part I and III (MDS-UPDRS I and III) and Montreal Cognitive Assessment (MoCA) data, the data beyond 7 years is sparse, particularly for participants with *LRRK2* and *GBA* PD. As such, we analysed the first 7 years (mean follow-up = 5.81 years) of MDS-UPDRS I and III and MoCA data, as well as 5 years (mean follow-up = 3.98 years) of dopamine transporter (DAT) imaging with single-photon emission computed tomography (DAT-SPECT) as measures of PD-related non-motor decline, motor decline, cognitive decline, and loss of DAT, respectively.^24^, ^25^, ^26^ Only “off” medication state MDS-UPDRS III assessments were included. The number of observations collected vs. time for MDS-UPDRS I and III, MoCA, and DAT-SPECT data is shown in Supplemental Figure S1. Based on the literature, we considered a difference in MDS-UPDRS III score of 4.63 points to be clinically meaningful.^27^

Cerebrospinal fluid (CSF) was collected from participants (n = 564) at baseline and analysed by the PPMI team using the 24-h (n = 59) and 150-h (n = 505) Amprion α-syn SAAs.^18^^,^^28^^,^^29^ While there may be interprotocol variability, we used data from both the 24-h and 150-h Amprion α-syn SAAs as the two protocols are overall comparable with the exception of timeframe. Of the 165 participants with results from both the 24-h and 150-h only 8 had discrepancies between their results and were excluded.

### Statistics

All participants with genome-wide sequencing data, a PD diagnosis, and baseline α-syn SAA results at the time of data access were included in our analysis. Our statistical analysis plan was developed *a priori* and refined *post hoc*. All statistical analyses were performed using SAS® software version 9.4, and all figures were created using R software version 4.4.1.

A linear mixed-effects model was used to estimate the yearly rate of change in MDS-UPDRS I and III score, MoCA score, and DAT-SPECT specific binding ratio (SBR) for α-syn SAA positive and negative participants. In addition, we categorized the 505 participants with 150-h α-syn SAA data into quartiles (Q0, Q1, Q2, and Q3) based on their α-syn SAA T50, TTT, AUC, and fluorescence maximum (Fmax) and estimated the yearly rate of change in MDS-UPDRS I and III score, MoCA score, and DAT-SPECT SBR for each quartile. Kinetic parameters were averaged across replicates. α-Syn SAA negative participants were automatically assigned to Q0. Given previously reported differences in progression between participants with sporadic, *LRRK2,* and *GBA* PD, as well as reported differences in α-syn SAA positivity and kinetic parameters, we estimated separate rates of progression for participants with sporadic, *LRRK2*, and *GBA* PD.^17^, ^18^, ^19^, ^20^, ^21^ Each outcome was modelled independently.

Models assumed participant-level random intercepts and slopes for MDS-UPDRS I and III, MoCA, and DAT-SPECT SBR with unstructured covariance and heteroscedastic variance between groups. All models were adjusted for baseline demographic characteristics (age, sex, race [two levels], ethnicity [two levels], and education [three levels] (all self-reported by participants)), time since original diagnosis at baseline, and baseline score, as well as their interaction with time as a fixed effect. We also adjusted for the levodopa equivalent daily dosage (LEDD) at the time of each visit. Estimates were obtained for an average participant at an LEDD of zero. A significance level of α = 0.05 was used for all analyses. With 514 SAA-positive and 72 SAA-negative participants, this study had 99% power at α = 0.05 to detect a 1.0 point/year change in MDS-UPDRS III score. Confounding variables were identified based on a review of existing literature and modelled according to a directed acyclic graph (DAG) (Supplemental Figure S2).^17^, ^18^, ^19^, ^20^^,^^30^ Exposure and adjustment variables were treated as fixed effects to account for their consistent influence across all participants. All model residuals were assessed by Q–Q plots for normality assumption, residuals vs. fitted plots for heteroscedastic assumption, and residuals vs. predictor plots for linearity assumption. The variance inflation factor (VIF) was used to check for multicollinearity, and variables with VIF >10 would result in revising the DAG and statistical model.

### Role of funders

The funders had no role in the study design, analysis, interpretation of data, or the writing of the report. While the funders were not directly involved in data collection, PPMI is funded by the Michael J. Fox Foundation and is associated with Aligning Science Across Parkinson's.

## Results

We analysed longitudinal clinical data from 564 PPMI participants, including 332 with sporadic PD, 162 with *LRRK2* PD, and 70 with *GBA* PD. Baseline demographic and clinical characteristics for each group categorized by α-syn SAA result are shown in Table 1. Interestingly, across sporadic *LRRK2* and *GBA* PD, α-syn SAA negative participants were older at disease onset (Table 1). Baseline demographic and clinical characteristics for each *LRRK2* variant categorized by α-syn SAA result are shown in Supplemental Table S1.

**Table 1:** Baseline demographic and clinical characteristics for sporadic, *LRRK2*, and *GBA* PD categorized by α-synuclein seed amplification assay result.

	Sporadic PD		*LRRK2* PD		*GBA* PD	
	Positive SAA	Negative SAA	Positive SAA	Negative SAA	Positive SAA	Negative SAA
N	315	17	111	51	66	4
Age at baseline (years)	61.8 (9.8)	67.2 (11.0)	59.6 (9.0)	68.1 (6.9)	61.4 (9.0)	66.3 (10.3)
Years since original diagnosis	0.33 (0.17, 0.75)	0.58 (0.42, 1.08)	1.92 (0.79, 3.87)	2.42 (1.17, 5.12)	1.17 (0.50, 3.67)	0.96 (0.52, 1.42)
Age at disease onset (years)	61.1 (9.78)	66.4 (10.9)	57.2 (9.2)	65.1 (7.39)	59.2 (9.22)	65.3 (10.1)
Male sex	208 (66.0)	11 (64.7)	63 (56.8)	20 (39.2)	40 (60.6)	3 (75.0)
Race						
White	283 (89.8)	17 (100)	100 (90.1)	43 (84.3)	63 (95.5)	4 (100)
Asian	8 (2.5)	0	0	0	0	0
Black	7 (2.2)	0	0	0	0	0
Multiracial	10 (3.2)	0	7 (6.3)	8 (15.7)	2 (3.0)	0
Other	5 (1.6)	0	3 (2.7)	0	1 (1.5)	0
Not Reported	2 (0.6)	0	1 (0.9)	0	0	0
Hispanic or Latino ethnicity	7 (2.2)	0	19 (17.1)	16 (31.4)	2 (3.0)	0
Education						
Less than 12 years	20 (6.3)	0	10 (9.0)	15 (29.4)	4 (6.1)	0
12–16 years	191 (60.6)	9 (52.9)	50 (45.0)	21 (41.2)	30 (45.5)	2 (50.0)
Greater than 16 years	104 (33.0)	8 (47.1)	51 (45.9)	15 (29.4)	32 (48.5)	2 (50.0)
Relatives with Parkinson's disease						
Parent	37 (11.7)	0	34 (30.6)	23 (45.1)	12 (18.2)	1 (25.0)
Other	55 (17.5)	0	37 (33.3)	25 (49.0)	13 (19.7)	0
Time to dopaminergic medication	1.58 (0.94, 2.58)	1.58 (0.92, 2.17)	NA	NA	NA	NA
MDS-UPDRS III score at baseline	20.1 (8.9)	19.1 (5.3)	20.1 (10.1)	17.5 (7.9)	25.2 (11.3)	20.0 (13.0)
MDS-UPDRS I score at baseline	4.25 (3.06)	6.29 (5.17)	5.62 (4.05)	6.12 (5.02)	5.68 (3.90)	2.75 (2.75)
MoCA score at baseline	27.2 (2.3)	26.8 (2.1)	26.6 (2.8)	25.1 (3.1)	26.4 (2.7)	26.2 (2.6)
DAT-SPECT SBR at baseline						
Caudate	1.94 (1.67, 2.33)	1.77 (1.51, 2.40)	1.91 (1.53, 2.29)	1.91 (1.50, 2.22)	1.81 (1.31, 2.26)	1.42 (1.22, 1.79)
Putamen	0.79 (0.65, 0.96)	0.78 (0.55, 1.31)	0.73 (0.58, 0.90)	0.82 (0.59, 1.04)	0.69 (0.56, 0.88)	0.46 (0.35, 1.03)

Data is shown as n (%), mean (standard deviation), or median (IQR). Statistical analysis comparing group characteristics was not performed.

Abbreviations: PD, Parkinson's disease; LRRK2, leucine-rich repeat kinase 2; GBA, glucocerebrosidase; MDS-UPDRS, Movement Disorder Society Unified Parkinson's Disease Rating Scale; MoCA, Montreal Cognitive Assessment; DAT-SPECT, dopamine transporter imaging with single-photon emission computed tomography; SBR, specific binding ratio.

**Fig. 1:**
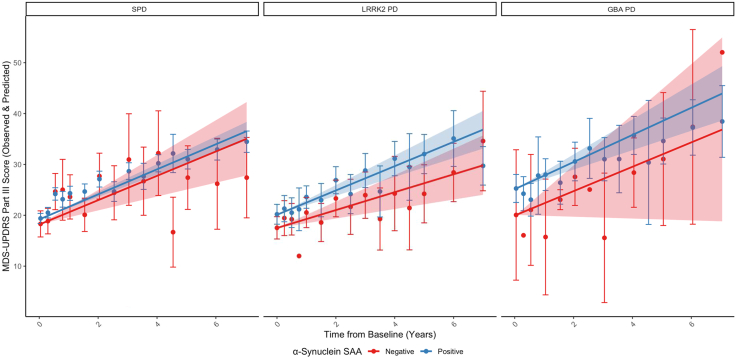
**Change in MDS-UPDRS III for participants with sporadic, *LRRK2*, and *GBA* PD categorized by α-synuclein seed amplification assay result**. Observed and predicted change in MDS-UPDRS III adjusted for age, time since diagnosis, sex, race, ethnicity, years of education, levodopa equivalent daily dosage, and baseline score in individuals with α-synuclein seed amplification assay positive and negative sporadic (n = 315 vs. 17), *LRRK2* (n = 111 vs. 51), and *GBA* PD (n = 66 vs. 4). Comparing the difference in change to those in the α-syn SAA negative group as reference. Data points represent the mean observed value at the corresponding time point. Lines represent the predicted value over time after adjustment. Error bars and shading represent 95% confidence intervals for observed and predicted values. Abbreviations: PD, Parkinson's disease; LRRK2, leucine-rich repeat kinase 2; GBA, glucocerebrosidase; α-synuclein SAA, α-synuclein seed amplification assay; MDS-UPDRS III, Movement Disorder Society Unified Parkinson's Disease Rating Scale Part III.

Participants with α-syn SAA positive sporadic PD (n = 315; MDS-UPDRS III points per year: 2.46 (95% confidence interval: 2.20–2.72)) progressed similarly to those with α-syn SAA negative sporadic PD (n = 17; 2.39 (1.36–3.42); difference = 0.07 (−0.99–1.12), p = 0.90) (Fig. 1 and Table 2). While the difference was not statistically significant, there was a trend toward faster motor decline in participants with α-syn SAA positive *LRRK2* PD (n = 111; 2.39 (1.86–2.92)) compared to those with α-syn SAA negative *LRRK2* PD (n = 51; 1.76 (0.93–2.60); difference = 0.63 (−0.29 to 1.55), p = 0.18). However, excluding R1441C/G + M1646T carriers (n = 4 SAA positive, 11 SAA negative) eliminated this trend (2.33 (1.79–2.86) vs. 2.26 (1.34–3.18); difference = 0.07 (−0.93 to 1.07, p = 0.89) (Table 3). Stratifying by individual *LRRK2* variants, α-syn SAA positive R1441C/G + M1646T carriers (n = 4; 3.89 (1.22–6.55)) progressed significantly faster than α-syn SAA negative R1441C/G + M1646T carriers (n = 11; 0.31 (−1.32 to 1.93); difference = 3.58 (0.56–6.60), p = 0.02). Based on a clinically meaningful difference of 4.63 points, there was no statistically significant or clinically meaningful difference between α-syn SAA positive G2019S carriers (n = 85; 2.29 (1.65–2.94)) and their α-syn SAA negative counterparts (n = 35; 2.18 (1.21–3.16); difference = 0.11 (−0.98 to 1.19), p = 0.85).^27^ Similarly, there was no statistically significant or clinically meaningful difference between α-syn SAA positive N2081D/N14D carriers (n = 22; 2.46 (1.38–3.55)) and their α-syn SAA negative counterparts (n = 5; 3.12 (0.26–5.99); difference = −0.66 (−3.72 to 2.39), p = 0.66). There was also no statistically significant or clinically meaningful difference between participants with α-syn SAA positive *GBA* PD (n = 66; 2.67 (1.91–3.44)) and those with α-syn SAA negative *GBA* PD (n = 4; 2.40 (−0.18 to 4.99); difference = 0.27 (−2.42 to 2.96), p = 0.84).

**Table 2:** Change in MDS-UPDRS III for participants with sporadic, *LRRK2*, and *GBA* PD categorized by α-synuclein seed amplification assay result.

Group	SAA result	MDS-UPDRS III rate of change	
		Slope/β (95% CI)	p-value
Sporadic	Negative	2.39 (1.36, 3.42)	
	Positive	2.46 (2.20, 2.72)	
	Positive vs. negative	0.07 (−0.99, 1.12)	0.90
*LRRK2*	Negative	1.76 (0.93, 2.60)	
	Positive	2.39 (1.86, 2.92)	
	Positive vs. negative	0.63 (−0.29, 1.55)	0.18
*GBA*	Negative	2.40 (−0.18, 4.99)	
	Positive	2.67 (1.91, 3.44)	
	Positive vs. negative	0.27 (−2.42, 2.96)	0.84
Overall SAA positive vs. negative		0.32 (−0.68, 1.33)	0.53
*GBA* vs. Sporadic	Negative	0.01 (−2.75, 2.78)	0.99
	Positive	0.22 (−0.58, 1.02)	0.59
*LRRK2* vs. Sporadic	Negative	−0.63 (−1.96, 0.70)	0.35
	Positive	−0.07 (−0.66, 0.53)	0.82

Slopes (unit per year) were estimated from linear mixed models. Fixed effects included time ∗ (genetic form ∗ SAA results, baseline MDS-UPDRS III, baseline age, years since original diagnosis to baseline, sex, race [two levels], ethnicity [two levels], and education [three levels]) and LEDD. Participant-level random intercepts and slopes with unstructured covariance, heteroscedastic by genetic form.

Abbreviations: PD, Parkinson's disease; LRRK2, leucine-rich repeat kinase 2; GBA, glucocerebrosidase; α-synuclein SAA, α-synuclein seed amplification assay; MDS-UPDRS III, Movement Disorder Society Unified Parkinson's Disease Rating Scale Part III.

**Table 3:** Change in MDS-UPDRS III for G2019S, R1441C/G + M1646T, and N2081D/N14D carriers categorized by α-synuclein seed amplification assay result.

Group	SAA result	MDS-UPDRS III rate of change	
		Slope/β (95% CI)	p-value
G2019S	Negative	2.18 (1.21, 3.16)	
	Positive	2.29 (1.65, 2.94)	
	Positive vs. negative	0.11 (−0.98, 1.19)	0.85
N2081D/N14D	Negative	3.12 (0.26, 5.99)	
	Positive	2.46 (1.37, 3.55)	
	Positive vs. negative	−0.66 (−3.72, 2.39)	0.66
R1441C/G + M1646T	Negative	0.31 (−1.32, 1.93)	
	Positive	3.89 (1.22, 6.55)	
	Positive vs. negative	3.58 (0.56, 6.60)	0.02
*LRRK2* (excluding R1441C/G + M1646T)	Negative	2.26 (1.34, 3.44)	
	Positive	2.33 (1.79, 2.86)	
	Positive vs. negative	0.07 (−0.93, 1.07)	0.89
*LRRK2* (excluding R1441C/G + M1646T) vs. Sporadic	Negative	−0.15 (−1.52, 1.22)	0.83
	Positive	−0.13 (−0.73, 0.47)	0.67

Slopes (unit per year) were estimated from linear mixed models. Fixed effects included time ∗ (genetic form ∗ SAA results, baseline MDS-UPDRS III, baseline age, years since original diagnosis to baseline, sex, race [two levels], ethnicity [two levels], and education [three levels]) and LEDD. Participant-level random intercepts and slopes with unstructured covariance, heteroscedastic by genetic form.

Abbreviations: PD, Parkinson's disease; LRRK2, leucine-rich repeat kinase 2; α-synuclein SAA, α-synuclein seed amplification assay; MDS-UPDRS III, Movement Disorder Society Unified Parkinson's Disease Rating Scale Part III.

Additionally, we obtained similar results in our comparison of the rate of change in MDS-UPDRS I scores (Supplemental Figure S3 and Supplemental Table S2). The rate of change in MoCA scores was limited in all participants (Supplemental Figure S4 and Supplemental Table S2). We also found no statistically significant difference in our comparison of the rate of DAT-SPECT SBR loss (Supplemental Figures S5 and S6 and Supplemental Table S2).

There was no statistically significant or clinically meaningful difference in the rate of motor decline between participants with α-syn SAA positive *LRRK2* PD and their α-syn SAA positive sporadic PD counterparts (difference = −0.07 (−0.66 to 0.53); p = 0.82). While there was no statistically significant difference, there was a trend towards slower motor decline in α-syn SAA negative *LRRK2* PD compared to those with α-syn SAA negative sporadic PD (difference = −0.63 (−1.96 to 0.70); p = 0.35) that seemed to be driven by R1441C/G + M1646T carriers (Tables 2 and 3). Similarly, there was no statistically significant or clinically meaningful difference between α-syn SAA positive (difference = 0.22 (−0.58 to 1.02); p = 0.59) or negative (difference = 0.01 (−2.75 to 2.78); p = 0.99) *GBA* PD and their sporadic PD counterparts.

To determine if α-syn SAA kinetic parameters are associated with PD progression, we categorized the 505 PPMI participants with 150-h α-syn SAA data into quartiles based on their average T50, TTT, AUC, and Fmax from three replicates. All α-syn SAA negative participants were assigned to Q0 (n = 63), and we categorized the remaining 442 α-syn SAA positive participants into equal tertiles (Q1 n = 147, Q2 n = 148, Q3 n = 147). Participants T50 ranged from 40 h to 121 h, TTT ranged from 43 h to 122 h, AUC ranged from 6.3E6 to 3.4E7, and Fmax ranged from 1.4E4 to 1.5E5. We found no statistically significant or clinically meaningful differences between participants in T50 Q0, Q1, Q2, and Q3 (Supplemental Table S3). Similarly, we found no clinically meaningful differences between participants in TTT, AUC, or Fmax Q0, Q1, Q2, and Q3 (Supplemental Tables S4, S5, and S6).

## Discussion

The development of α-syn SAAs has transformed our ability to detect α-syn seeds and diagnose PD.^7^^,^^21^^,^^28^^,^^29^ Making use of MDS-UPDRS I and III, MoCA, DAT-SPECT, and baseline α-syn SAA data from the PPMI cohort, we found that there was no statistically significant association between baseline α-syn seeding activity, α-syn SAA kinetic parameters, and disease progression in sporadic, *LRRK2*, and *GBA* PD. Notably, there was a trend toward faster motor decline in α-syn SAA positive *LRRK2* PD compared to α-syn SAA negative *LRRK2* PD (p = 0.18), which was driven by R1441C/G + M1646T carriers.

In the sporadic PD group, the overwhelming majority of participants were α-syn SAA positive (315/332 [95%]), potentially reflecting the central role of α-syn aggregation in PD pathophysiology. While prior studies have suggested that α-syn SAA negativity may indicate an atypical or misdiagnosed form of PD, our analysis did not reveal any clinically meaningful differences in their progression rates.^9^, ^10^, ^11^^,^^27^ Given the limited number of α-syn SAA-negative participants, this finding should be interpreted with caution. Notably, the upper bound of the confidence interval indicates that participants with α-syn SAA positive sporadic PD may progress up to 1 point per year faster than their α-syn SAA negative counterparts. Nevertheless, based on a minimal clinically important difference of 4.63 points, our results are still sufficiently precise to exclude any clinically meaningful difference over at least 4 years based on the upper bound estimate of 1 point per year.^27^ Future studies with larger sample sizes and additional biomarkers, such as tau or amyloid pathology, may help clarify whether α-syn SAA negative sporadic PD represents a distinct PD subtype or are a result of technical limitations in the sensitivity of the assay.

Similarly, we found no statistically significant differences in progression rates for *GBA* PD between α-syn SAA positive and negative participants. Due to the few α-syn SAA negative *GBA* PD participants in the cohort (4/70 [6%]), our estimates yielded wide confidence intervals and were not sufficiently precise to exclude a clinically meaningful difference over 2 years.^27^ However, the estimated difference was quite modest. Given that *GBA* mutations are strongly associated with α-syn pathology, α-syn SAA positivity may be a less distinguishing factor in this population.^18^^,^^31^

The proportion of α-syn SAA positive individuals in *LRRK2* PD was substantially lower than in sporadic PD (111/162 [69%] vs. 315/332 [95%]), providing more balanced sample sizes between α-syn SAA positive and negative groups within *LRRK2* PD.^21^ Our analysis found that participants with α-syn SAA positive *LRRK2* PD exhibited a trend towards faster rate of motor decline than their α-syn SAA negative counterparts. This trend was primarily driven by R1441C/G + M1646T carriers, among which α-syn SAA positive individuals progressed significantly faster than their α-syn SAA negative counterparts. The upper bound of the confidence interval indicates that participants with α-syn SAA positive *LRRK2* PD may progress up to 1.5 points per year faster than their α-syn SAA negative counterparts, excluding any clinically meaningful difference over at least 3 years. Additionally, there was a trend toward slower motor decline in α-syn SAA negative *LRRK2* PD compared to α-syn SAA negative sporadic PD, which was again driven by R1441C/G + M1646T carriers. A similar trend towards slower cognitive decline was observed in *LRRK2* PD compared to sporadic PD. These findings, in general, align with previous studies indicating that *LRRK2* PD may progress slower.^17^^,^^19^^,^^20^ Future investigations should address whether the lower rates of α-syn SAA positivity and slower disease progression observed in *LRRK2* PD are related, and especially whether R1441C/G + M1646T may be associated with distinct progression patterns.^17^^,^^19^^,^^21^^,^^32^

While previous studies on patients with PD and Lewy Body Dementia have shown that α-syn SAA kinetic parameters may be associated with cognitive decline, we did not find any statistically significant or clinically meaningful association between T50, TTT, AUC, or Fmax and PD motor and cognitive progression.^9^^,^^10^ The differences between our results and those of the previous studies may be explained by differences in α-syn SAA protocols, statistical methods, and cohorts. For example, the rate of change in MoCA scores was limited in all participants in the PPMI cohort potentially masking any differences in cognitive decline. While there is evidence that T50, TTT, AUC, and Fmax may be associated with the number and concentration of α-syn seeds, how that association translates to α-syn pathology and clinical outcomes remains to be determined.^33^, ^34^, ^35^ Robust and direct quantitative measures of α-syn seeds may help define the relationship between α-syn pathology and PD progression.

Here we leveraged rigorously assembled longitudinal and α-syn SAA data from the PPMI cohort along with a statistical model that stringently accounts for variability in disease progression both within and between participants. Overall, our findings suggest that α-syn SAA positivity is not a robust predictor of PD progression across clinical and imaging measures in this cohort. However, the absence of significant findings does not imply a lack of biological relevance given the limitations of this study. Despite the overall high quality of the PPMI dataset, there was substantial person-to-person variability in progression rates that current prognostics cannot predict. While some clinically meaningful differences could be excluded, the precision of our estimates was limited by the small number of α-syn SAA negative participants. Further, compared to the overall course of PD, a 7-year follow-up period may not be long enough to accurately address PD progression. Moreover, α-syn pathology in brain tissues may be influenced by factors beyond what is detectable in CSF collected through lumbar puncture. Additionally, while we adjusted for key confounding variables, there may still be other confounders, e.g., co-existing medical conditions and lifestyle factors, that may contribute to PD progression. Lastly, the cohort primarily contains participants of European descent, and, as such, our results may not be generalizable to non-European populations.

Our study provides fresh insight into the relationship between α-syn SAAs and PD progression. While we found no statistically significant association between α-syn SAA positivity and the rate of PD progression, the relationships among baseline α-syn seeding activity, disease heterogeneity, disease stage, and PD progression warrant further investigation. Importantly, participants with α-syn SAA negative PD still exhibit symptomatic progression, and the presence of α-syn seeding activity at baseline may not fully capture the dynamic nature of disease progression and neurodegeneration. As such, future studies should investigate longitudinal changes of α-syn seeding activity together with complementary biomarkers for PD progression, including among pre-manifest individuals.

## Contributors

Conceptualization: JGS, XYZ, EAM, JW, AB, JMD, HW, MAS, MC, XHZ, and XC; Data Curation: JGS and XYZ; Methodology, Software, and Formal Analysis: JGS, XYZ, EAM, JW, JMD, HW, MAS, MC, XHZ, and XC; Visualization: JGS, and XYZ; Data Validation: JGS, XYZ, EAM, JW, AB, MC, and XC; Writing—Original Draft: JGS, XYZ, and XC; Writing—Review & Editing: JGS, XYZ, EAM, JW, AB, JMD, HW, MAS, MC, XHZ, and XC; Supervision: EAM, MAS, MC, XHZ, and XC; Funding Acquisition: XC. JS, XYZ, EAM, JW, AB, MC, and XC have all had direct access to and have verified the underlying data. All authors have read and approved the final version of the manuscript.

## Data sharing statement

Data used in the preparation of this article is openly available to qualified researchers, and in the case of whole genome sequencing data, it is available upon request. Data were obtained in June 2024 from the Parkinson's Progression Markers Initiative (PPMI) database (www.ppmi-info.org/access-data-specimens/download-data, RRID:SCR00_6431). For up-to-date information on the study, visit www.ppmi-info.org. All other resources and codes used in this study can be found at https://doi.org/10.5281/zenodo.14984600.

## Declaration of interests

JGS, XYZ, JW, AB, JMD, MAS, MC, XHZ, and XC declare no conflicts of interest.

EAM received payments to his institution from AI Therapeutics, Biohaven, Calico Therapeutics, Denali Therapeutics, ITB-Med, Janssen, Lilly, Mitsubishi Tanabe Pharma America, Neurizon, Prilenia Therapeutics, Revalesio, Seelos Therapeutics, UCB/Ra Pharma, Woolsey. He received compensation for serving on scientific advisory boards for Annexon, Bial Biotech, Chase Therapeutics, HillHurst, Merck; for serving on steering committees for Biogen, UCB; and for serving on data monitoring committees for Argenx, NeuroSense, Novartis, Sanofi.

HW received payments from Takeda Pharmaceuticals, AbbVie, Kyowa Kirin, Sumitomo Pharma, Eisai, and Ono Corporation for work unrelated to this study.

## Appendix A Supplementary data

Supplemental Tables

Caption for Supplementary Material
