# Supplementary figures and images for: Baseline α-synuclein seeding activity and disease progression in sporadic and genetic Parkinson's disease in the PPMI cohort
Source: eBioMedicine. 2025 Aug 6;119:105866. doi: 10.1016/j.ebiom.2025.105866 (PMC12354789; doi:10.1016/j.ebiom.2025.105866)

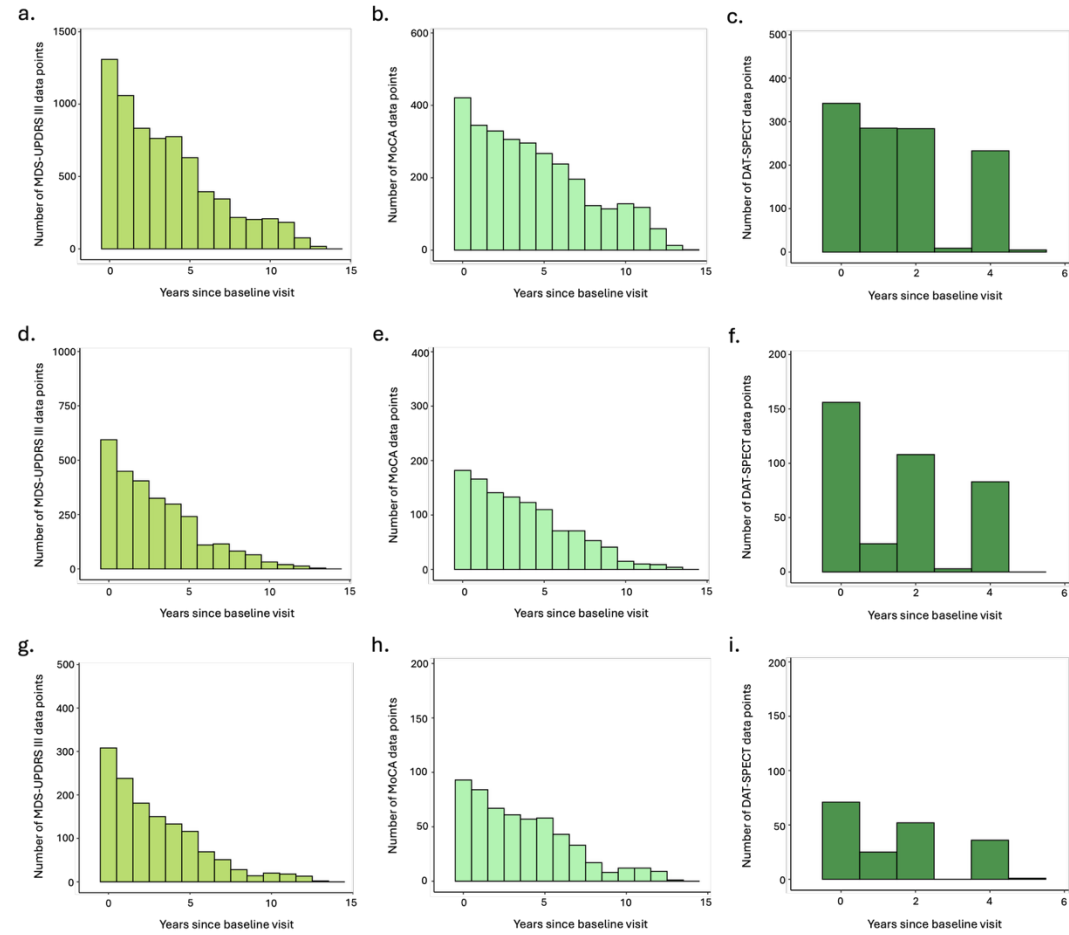

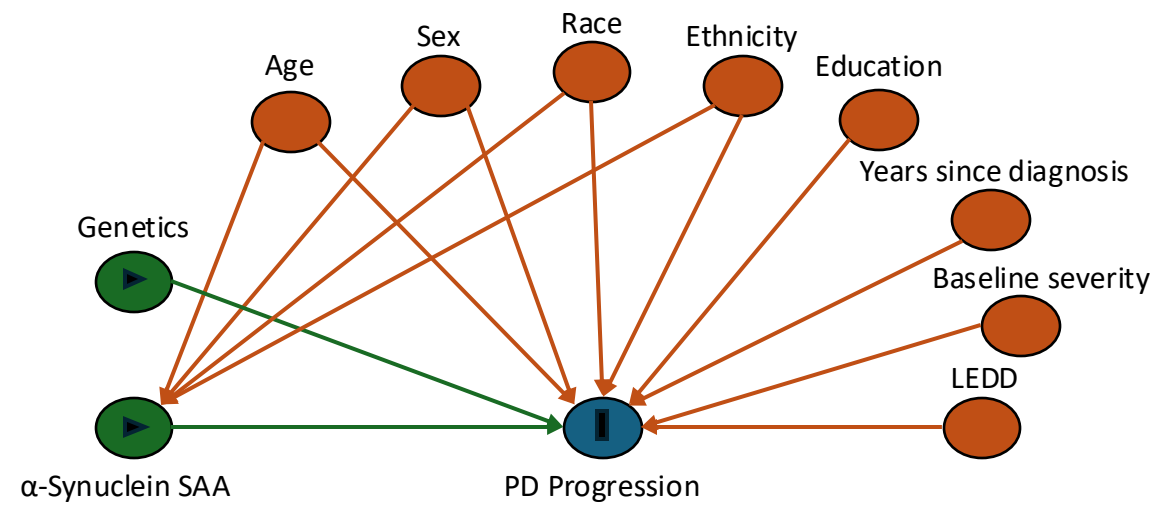

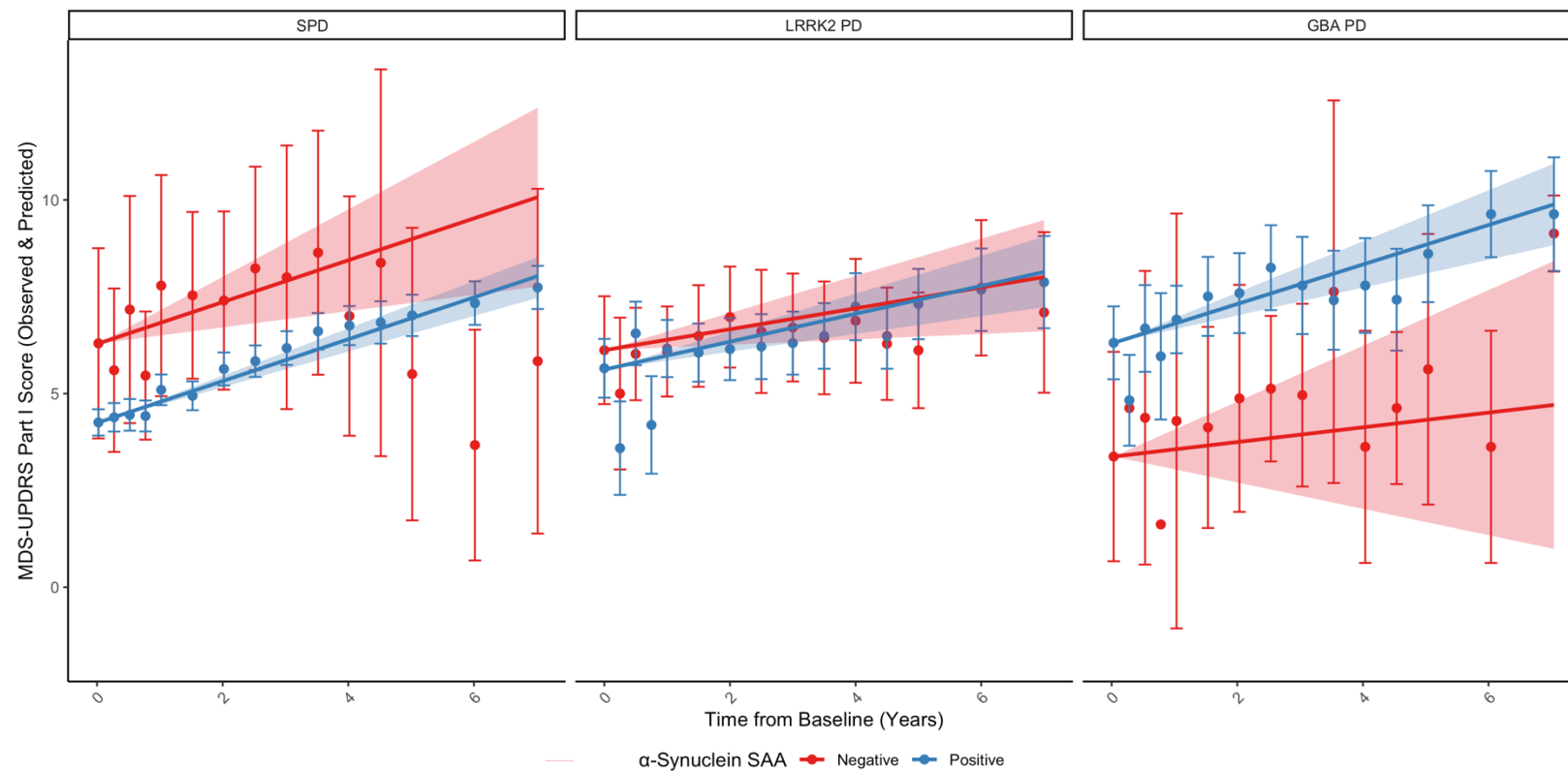

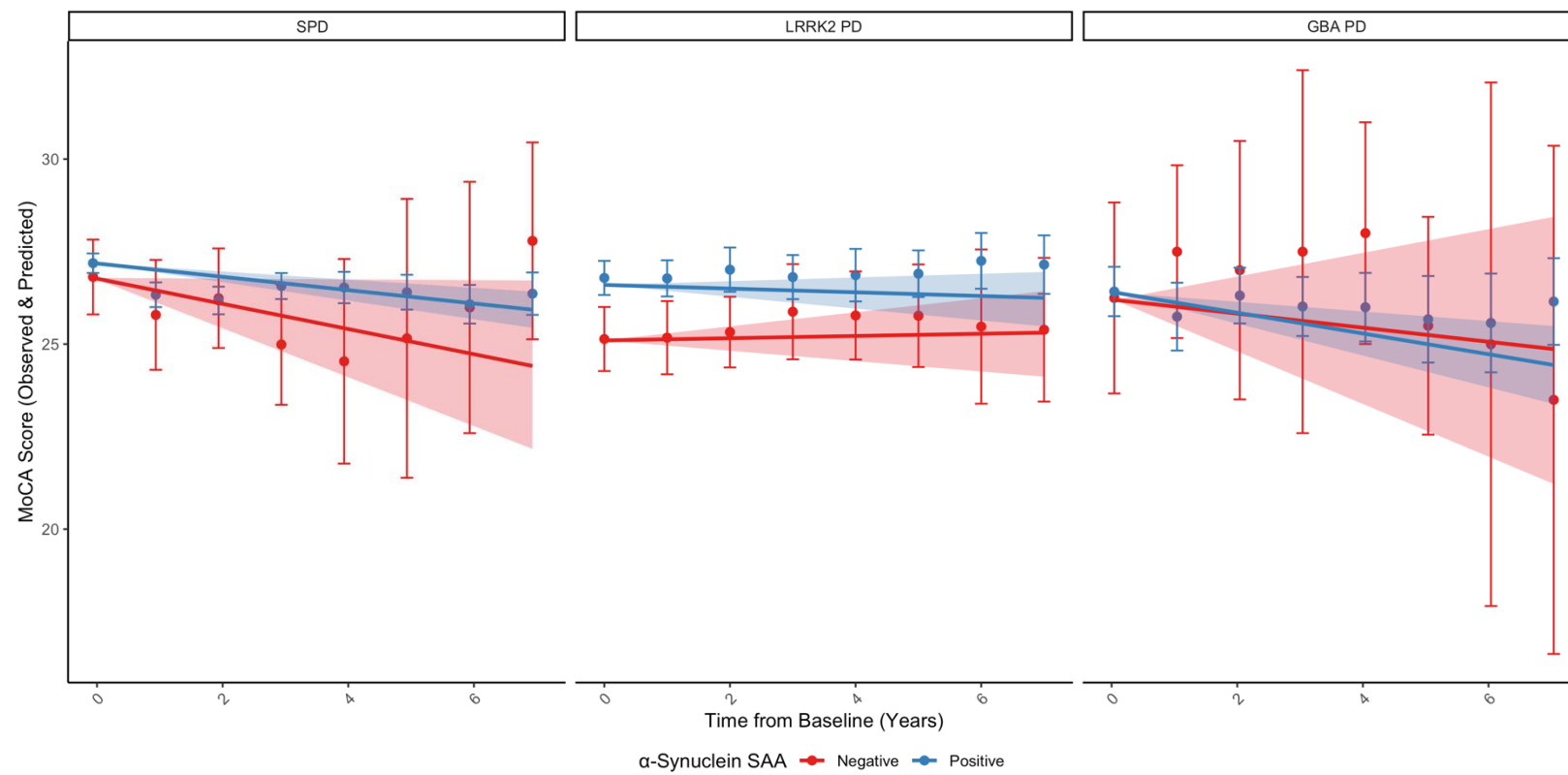

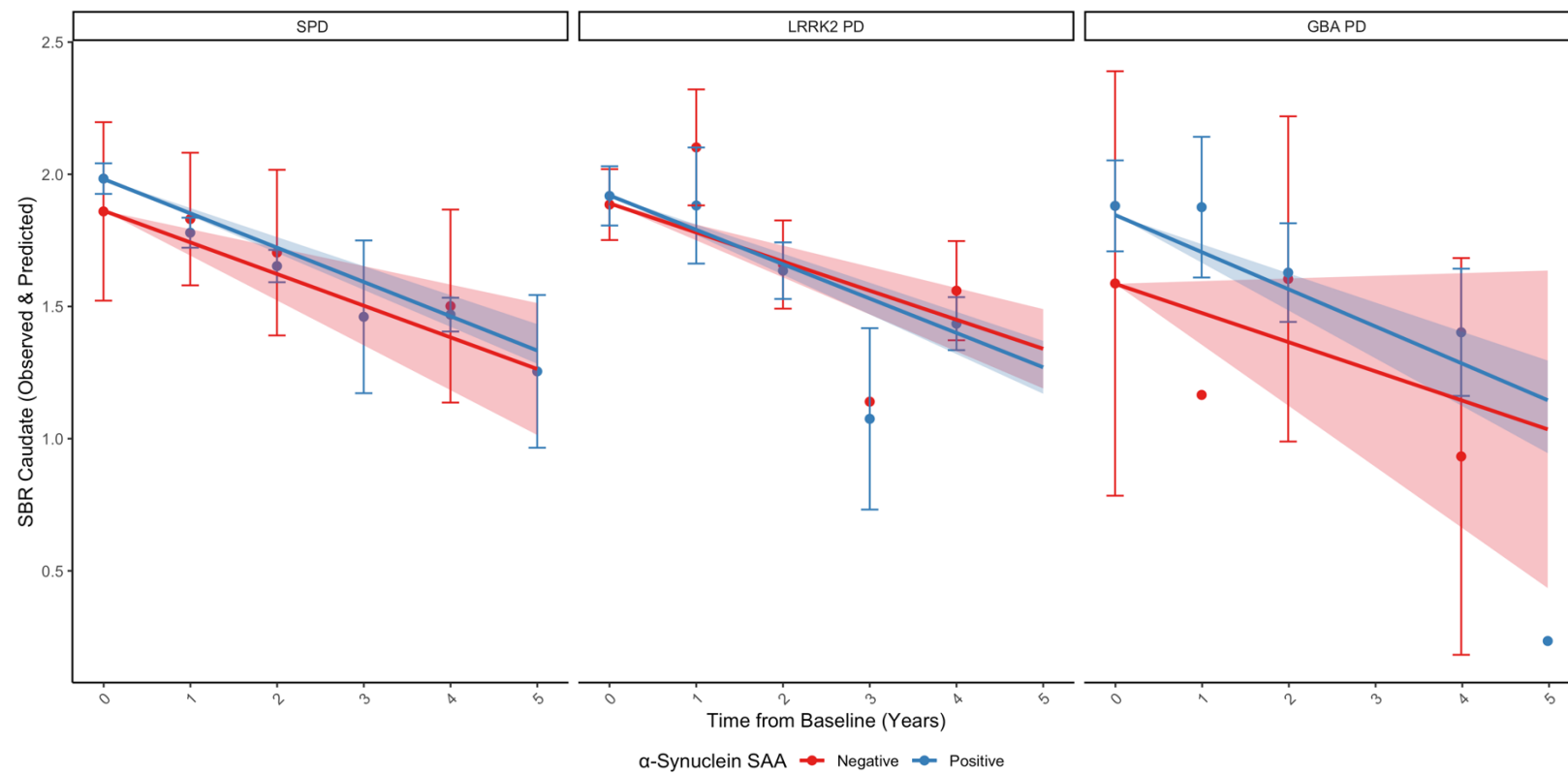

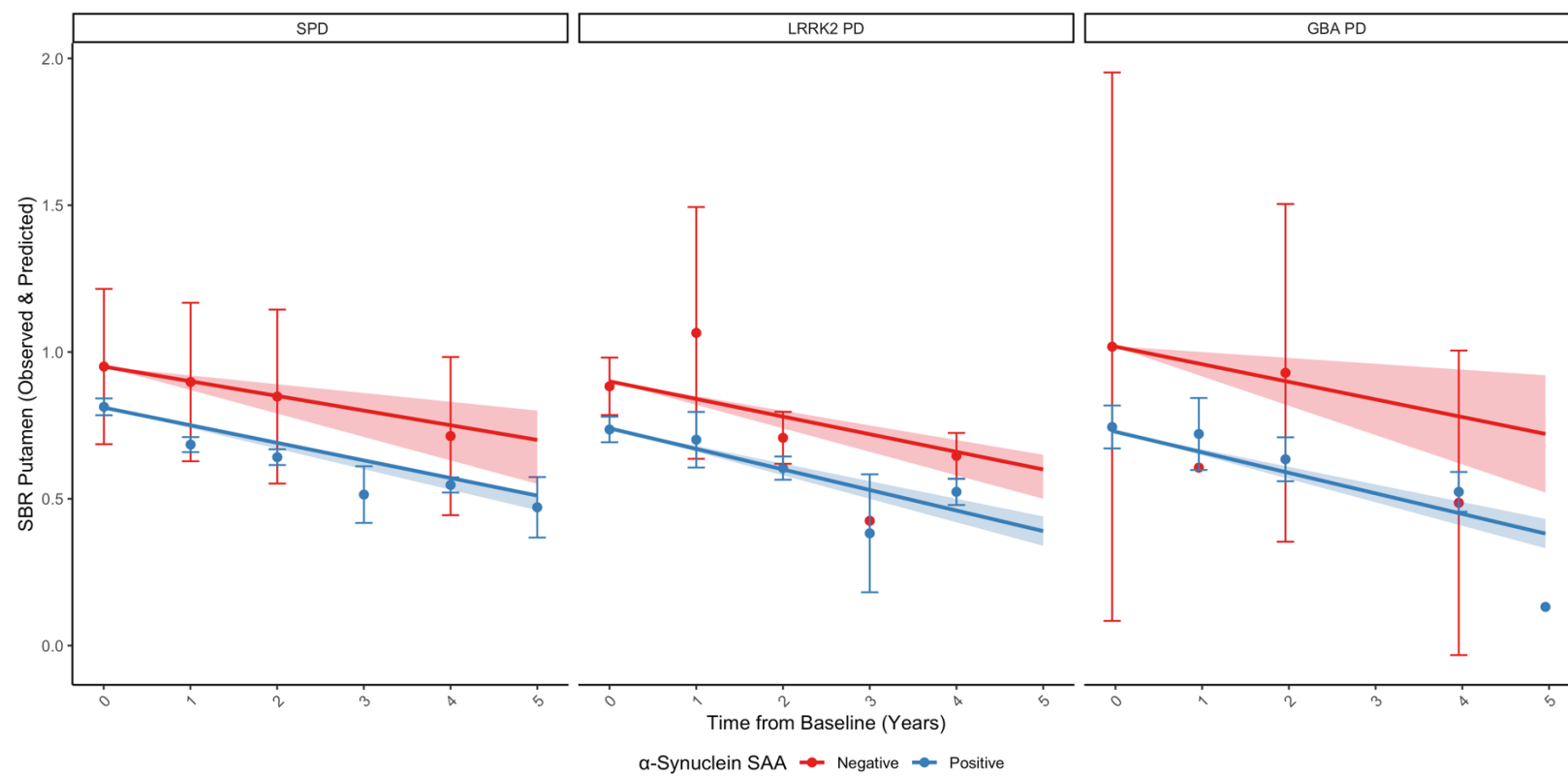

Supplement: Supplemental Figures [file mmc1.pdf]
