# Supplementary material for: Baseline α-synuclein seeding activity and disease progression in sporadic and genetic Parkinson's disease in the PPMI cohort
Source: eBioMedicine. 2025 Aug 6;119:105866. doi: 10.1016/j.ebiom.2025.105866 (PMC12354789; doi:10.1016/j.ebiom.2025.105866)
Supplement: Supplemental Tables [file mmc2.docx]

|  | **G2019S** | | **N2081D/N14D** | | **R1441C/G+M1646T** | |
| --- | --- | --- | --- | --- | --- | --- |
|  | **Positive SAA** | **Negative SAA** | **Positive SAA** | **Negative SAA** | **Positive SAA** | **Negative SAA** |
| N | 85 | 35 | 22 | 5 | 4 | 11 |
| Age at baseline (years) | 59.6 (8.7) | 68.7 (5.9) | 59.7 (9.4) | 67.4 (11.2) | 58.5 (15.2) | 66.5 (8.0) |
| Years since original diagnosis | 2.80 (1.08, 4.33) | 2.67 (0.92, 3.87) | 0.97 (0.33, 1.54) | 4.70 (5.25, 5.58) | 2.52 (0.96, 3.19) | 3.15 (1.50, 5.00) |
| Age at disease onset (years) | 56.8 (8.8) | 66.0 (6.23) | 58.7 (9.7) | 62.7 (11.1) | 55.9 (17.4) | 63.3 (9.08) |
| Male sex | 47 (55.3) | 16 (45.7) | 14 (63.6) | 1 (20.0) | 2 (50.0) | 3 (27.3) |
| Race |  |  |  |  |  |  |
| White | 77 (90.6) | 33 (94.3) | 21 (95.5) | 5 (100) | 2 (50) | 5 (45.5) |
| Asian | 0 | 0 | 0 | 0 | 0 | 0 |
| Black | 0 | 0 | 0 | 0 | 0 | 0 |
| Multiracial | 4 (4.7) | 2 (5.7) | 1 (4.5) | 0 | 2 (50) | 6 (54.5) |
| Other | 3 (3.5) | 0 | 0 | 0 | 0 | 0 |
| Not Reported | 1 (1.2) | 0 | 0 | 0 | 0 | 0 |
| Hispanic or Latino ethnicity | 16 (18.8) | 7 (20.0) | 0 | 0 | 3 (75) | 9 (81.8) |
| Education |  |  |  |  |  |  |
| Less than 12 years | 8 (9.41) | 8 (22.9) | 1 (4.5) | 0 | 1 (25) | 7 (63.6) |
| 12-16 years | 35 (41.2) | 14 (40.0) | 13 (59.1) | 4 (80) | 2 (50) | 3 (27.3) |
| Greater than 16 years | 42 (49.4) | 13 (37.1) | 8 (36.4) | 1 (20) | 1 (25) | 1 (9.1) |
| Relatives with Parkinson’s disease |  |  |  |  |  |  |
| Parent | 31 (36.5) | 16 (45.7) | 1 (4.5) | 2 (40) | 2 (50) | 5 (45.5) |
| Other | 29 (34.1) | 16 (45.7) | 5 (22.7) | 1 (20) | 3 (75) | 8 (72.7) |
| MDS-UPDRS III score at baseline | 20.6 (10.7) | 18.2 (8.8) | 17.4 (7.4) | 21.4 (6.2) | 17.3 (4.8) | 13.9 (3.8) |
| MDS-UPDRS I score at baseline | 6.1 (3, 9) | 6.5 (2, 9) | 4.1 (2, 6) | 7.2 (3, 10) | 3.7 (3, 4.5) | 4.2 (1, 4.5) |
| MoCA score at baseline | 26.7 (25.5, 28.5) | 25.6 (24, 27.5) | 26.9 (26, 28) | 27.8 (28, 28) | 28 (27.3, 29.3) | 22.6 (20.5, 25) |
| DAT-SPECT SBR at baseline |  |  |  |  |  |  |
| Caudate | 1.88 (1.43, 2.31) | 1.89 (1.47, 2.29) | 2.07 (1.58, 2.48) | 1.61 (1.39, 1.79) | 1.84 (1.78, 1.97) | 1.98 (1.73, 2.29) |
| Putamen | 0.72 (0.55, 0.85) | 0.91 (0.59, 1.07) | 0.81 (0.58, 1.05) | 0.64 (0.49, 0.76) | 0.67 (0.55, 0.80) | 0.88 (0.69, 1.10) |

**Supplemental Table 1. Baseline demographic and clinical characteristics for G2019S, N2081D/N14D, and R1441C/G+M1646T carriers categorized by α-synuclein seed amplification assay result**

Data is shown as n (%), mean (standard deviation), or median (IQR). Statistical analysis comparing group characteristics was not performed.

Abbreviations: PD, Parkinson's disease; MDS-UPDRS, Movement Disorder Society Unified Parkinson's Disease Rating Scale; MoCA, Montreal Cognitive Assessment; DAT-SPECT, dopamine transporter imaging with single-photon emission computed tomography; SBR, specific binding ratio.

|  |  | **MDS-UPDRS I** |  | **MoCA** |  | **Caudate SBR** |  | **Putamen SBR** |  |
| --- | --- | --- | --- | --- | --- | --- | --- | --- | --- |
| **Group** | **SAA** | **Slope/β (95% CI)** | **p-value** | **Slope/β (95% CI)** | **p-value** | **Slope/β (95% CI)** | **p-value** | **Slope/β (95% CI)** | **p-value** |
| Sporadic | Negative | 0.54 (0.21, 0.87) |  | -0.34 (-0.66, -0.01) |  | -0.12 (-0.17, -0.07) |  | -0.05 (-0.08, -0.03) |  |
|  | Positive | 0.54 (0.46, 0.61) |  | -0.18 (-0.25, -0.11) |  | -0.13 (-0.14, -0.11) |  | -0.06 (-0.07, -0.06) |  |
|  | Positive vs. negative | 0.00 (-0.34, 0.33) | 0.99 | 0.16 (-0.17, 0.48) | 0.35 | -0.01 (-0.06, 0.04) | 0.78 | -0.01 (-0.04, 0.01) | 0.33 |
| *LRRK2* | Negative | 0.27 (0.07, 0.48) |  | 0.03 (-0.14, 0.19) |  | -0.11 (-0.14, -0.08) |  | -0.06 (-0.08, -0.05) |  |
|  | Positive | 0.36 (0.23, 0.49) |  | -0.05 (-0.16, 0.05) |  | -0.13 (-0.15, -0.11) |  | -0.07 (-0.08, -0.06) |  |
|  | Positive vs. negative | 0.08 (-0.15, 0.31) | 0.48 | -0.08 (-0.27, 0.10) | 0.37 | -0.02 (-0.06, 0.02) | 0.27 | -0.01 (-0.03, 0.01) | 0.57 |
| *GBA* | Negative | 0.19 (-0.34, 0.72) |  | -0.19 (-0.71, 0.32) |  | -0.11 (-0.23, 0.01) |  | -0.06 (-0.10, -0.02) |  |
|  | Positive | 0.51 (0.36, 0.66) |  | -0.28 (-0.43, -0.13) |  | -0.14 (-0.18, -0.11) |  | -0.07 (-0.08, -0.06) |  |
|  | Positive vs. negative | 0.32 (-0.23, 0.87) | 0.25 | -0.09 (-0.62, 0.45) | 0.75 | -0.03 (-0.16, 0.09) | 0.62 | -0.01 (-0.05, 0.03) | 0.66 |
| Overall SAA positive vs. negative | | 0.13 (-0.09, 0.36) | 0.25 | 0 (-0.22, 0.22) | 0.99 | -0.02 (-0.07, 0.03) | 0.39 | -0.01 (-0.03, 0.01) | 0.30 |
|  |  |  |  |  |  |  |  |  |  |
| *GBA* vs. sporadic | Negative | -0.35 (-0.97, 0.27) | 0.26 | 0.14 (-0.46, 0.74) | 0.64 | 0.01 (-0.12, 0.14) | 0.90 | -0.01 (-0.05, 0.04) | 0.76 |
| *GBA* vs. sporadic | Positive | -0.03 (-0.19, 0.14) | 0.74 | -0.10 (-0.26, 0.06) | 0.22 | -0.02 (-0.05, 0.02) | 0.36 | 0 (-0.02, 0.01) | 0.53 |
| *LRRK2* vs. sporadic | Negative | -0.27 (-0.65, 0.12) | 0.18 | 0.36 (0.01, 0.72) | 0.047 | 0.01 (-0.05, 0.07) | 0.77 | -0.01 (-0.04, 0.02) | 0.45 |
| *LRRK2* vs. sporadic | Positive | -0.18 (-0.33, -0.03) | 0.022 | 0.13 (0, 0.25) | 0.05 | -0.01 (-0.03, 0.02) | 0.70 | -0.01 (-0.02, 0.01) | 0.47 |

**Supplemental Table 2: Change in MDS-UPDRS I, MoCA, and DAT-SPECT SBR for participants with sporadic, LRRK2, and GBA PD categorized by α-synuclein seed amplification assay result**

Slopes (unit per year) were estimated from linear mixed models. Fixed effects included time * (genetic form * SAA results, baseline measure for each corresponding marker, baseline age, years since original diagnosis to baseline, sex, race [two levels], ethnicity [two levels], and education [three levels]) and LEDD. Participant-level random intercepts and slopes with unstructured covariance, heteroscedastic by genetic form.

Abbreviations: PD, Parkinson's disease; LRRK2, leucine-rich repeat kinase 2; GBA, glucocerebrosidase; MDS-UPDRS, Movement Disorder Society Unified Parkinson's Disease Rating Scale; MoCA, Montreal Cognitive Assessment; DAT-SPECT, dopamine transporter imaging with single-photon emission computed tomography; SBR, specific binding ratio.

|  |  | **MDS-UPDRS III** |  | **MDS-UPDRS I** |  | **MoCA** |  | **Caudate SBR** |  | **Putamen SBR** |  |
| --- | --- | --- | --- | --- | --- | --- | --- | --- | --- | --- | --- |
| **Group** | **T50 Quartile** | **Slope/β (95% CI)** | **p-value** | **Slope/β (95% CI)** | **p-value** | **Slope/β (95% CI)** | **p-value** | **Slope/β (95% CI)** | **p-value** | **Slope/β (95% CI)** | **p-value** |
| Sporadic | Q0 | 2.47 (1.99, 2.94) |  | 0.43 (0.29, 0.56) |  | -0.19 (-0.30, -0.07) |  | -0.11 (-0.13, -0.09) |  | -0.07 (-0.08, -0.05) |  |
|  | Q1 | 2.33 (1.89, 2.76) |  | 0.55 (0.43, 0.67) |  | -0.16 (-0.26, -0.06) |  | -0.12 (-0.14, -0.09) |  | -0.06 (-0.07, -0.05) |  |
|  | Q1 vs. Q0 | -0.14 (-0.76, 0.48) | 0.66 | 0.13 (-0.05, 0.30) | 0.15 | 0.03 (-0.12, 0.18) | 0.71 | -0.01 (-0.03, 0.02) | 0.58 | 0.01 (-0.01, 0.02) | 0.27 |
|  | Q2 | 2.32 (1.88, 2.76) |  | 0.57 (0.44, 0.69) |  | -0.29 (-0.39, -0.18) |  | -0.14 (-0.16, -0.12) |  | -0.06 (-0.08, -0.05) |  |
|  | Q2 vs. Q0 | -0.15 (-0.77, 0.47) | 0.64 | 0.14 (-0.04, 0.32) | 0.12 | -0.09 (-0.25, 0.06) | 0.21 | -0.03 (-0.06, -0.00) | 0.04 | 0.00 (-0.01, 0.01) | 0.87 |
|  | Q3 | 2.90 (1.92, 3.89) |  | 0.62 (0.32, 0.92) |  | -0.34 (-0.61, -0.07) |  | -0.14 (-0.19, -0.09) |  | -0.05 (-0.08, -0.03) |  |
|  | Q3 vs. Q0 | 0.43 (-0.65, 1.52) | 0.43 | 0.19 (-0.14, 0.52) | 0.26 | -0.16 (-0.45, 0.13) | 0.29 | -0.03 (-0.08, 0.02) | 0.25 | 0.01 (-0.01, 0.04) | 0.39 |
| *LRRK2* | Q0 | 2.11 (1.27, 2.94) |  | 0.31 (0.09, 0.53) |  | -0.09 (-0.27, 0.07) |  | -0.12 (-0.16, -0.08) |  | -0.07 (-0.09, -0.05) |  |
|  | Q1 | 2.82 (1.86, 3.78) |  | 0.44 (0.17, 0.72) |  | -0.21 (-0.41, -0.02) |  | -0.12 (-0.16, -0.08) |  | -0.07 (-0.09, -0.05) |  |
|  | Q1 vs. Q0 | 0.72 (-0.50, 1.93) | 0.25 | 0.13 (-0.21, 0.47) | 0.44 | -0.12 (-0.36, 0.13) | 0.36 | -0.01 (-0.06, 0.05) | 0.85 | 0.00 (-0.03, 0.03) | 0.78 |
|  | Q2 | 2.54 (1.58, 3.49) |  | 0.38 (0.12, 0.65) |  | 0.01 (-0.19, 0.21) |  | -0.16 (-0.20, -0.11) |  | -0.07 (-0.09, -0.05) |  |
|  | Q2 vs. Q0 | 0.43 (-0.80, 1.66) | 0.49 | 0.07 (-0.26, 0.41) | 0.67 | 0.11 (-0.14, 0.37) | 0.39 | -0.04 (-0.09, 0.02) | 0.19 | 0.00 (-0.03, 0.03) | 0.91 |
|  | Q3 | 2.67 (1.91, 3.44) |  | 0.33 (0.08, 0.58) |  | -0.09 (-0.29, 0.09) |  | -0.14 (-0.19, -0.09) |  | -0.08 (-0.10, -0.05) |  |
|  | Q3 vs. Q0 | 0.34 (-0.89, 1.57) | 0.58 | 0.01 (-0.31, 0.33) | 0.94 | 0.00 (-0.25, 0.25) | 0.99 | -0.02 (-0.08, 0.03) | 0.43 | -0.01 (-0.04, 0.02) | 0.59 |
| *GBA* | Q0 | 2.53 (1.09, 3.97) |  | 0.46 (0.18, 0.74) |  | -0.29 (-0.53, -0.05) |  | -0.12 (-0.18, -0.06) |  | -0.06 (-0.08, -0.04) |  |
|  | Q1 | 2.06 (0.73, 3.39) |  | 0.59 (0.32, 0.86) |  | -0.21 (-0.42, 0.01) |  | -0.13 (-0.18, -0.08) |  | -0.07 (-0.09, -0.06) |  |
|  | Q1 vs. Q0 | -0.48 (-2.41, 1.46) | 0.62 | 0.13 (-0.25, 0.52) | 0.49 | 0.08 (-0.24, 0.39) | 0.61 | -0.02 (-0.09, 0.06) | 0.70 | -0.01 (-0.04, 0.02) | 0.41 |
|  | Q2 | 3.19 (1.78, 4.60) |  | 0.49 (0.19, 0.77) |  | -0.35 (-0.59, -0.10) |  | -0.19 (-0.25, -0.14) |  | -0.08 (-0.09, -0.06) |  |
|  | Q2 vs. Q0 | 0.66 (-1.33, 2.65) | 0.51 | 0.03 (-0.37, 0.43) | 0.88 | -0.06 (-0.40, 0.28) | 0.71 | -0.08 (-0.16, 0.00) | 0.06 | -0.02 (-0.04, 0.01) | 0.19 |
|  | Q3 | 2.98 (0.04, 5.92) |  | 0.21 (-0.39, 0.81) |  | -0.09 (-0.59, 0.40) |  | -0.15 (-0.28, -0.02) |  | -0.10 (-0.15, -0.05) |  |
|  | Q3 vs. Q0 | 0.45 (-2.83, 3.72) | 0.78 | -0.25 (-0.91, 0.41) | 0.45 | 0.19 (-0.36, 0.74) | 0.48 | -0.03 (-0.18, 0.11) | 0.67 | -0.04 (-0.09, 0.01) | 0.15 |

**Supplemental Table 3: Change in MDS-UPDRS I, MoCA, and DAT-SPECT SBR for participants with sporadic, LRRK2, and GBA PD categorized by time to 50% threshold quartile**

Slopes (unit per year) were estimated from linear mixed models. Fixed effects included time * (genetic form * SAA results, baseline measure for each corresponding marker, baseline age, years since original diagnosis to baseline, sex, race [two levels], ethnicity [two levels], and education [three levels]) and LEDD. Participant-level random intercepts and slopes with unstructured covariance, heteroscedastic by genetic form.

Abbreviations: PD, Parkinson's disease; T50, time to 50% threshold; LRRK2, leucine-rich repeat kinase 2; GBA, glucocerebrosidase; MDS-UPDRS, Movement Disorder Society Unified Parkinson's Disease Rating Scale; MoCA, Montreal Cognitive Assessment; DAT-SPECT, dopamine transporter imaging with single-photon emission computed tomography; SBR, specific binding ratio.

|  |  | **MDS-UPDRS III** |  | **MDS-UPDRS I** |  | **MoCA** |  | **Caudate SBR** |  | **Putamen SBR** |  |
| --- | --- | --- | --- | --- | --- | --- | --- | --- | --- | --- | --- |
| **Group** | **TTT Quartile** | **Slope/β (95% CI)** | **p-value** | **Slope/β (95% CI)** | **p-value** | **Slope/β (95% CI)** | **p-value** | **Slope/β (95% CI)** | **p-value** | **Slope/β (95% CI)** | **p-value** |
| Sporadic | Q0 | 2.13 (1.69, 2.57) |  | 0.54 (0.42, 0.67) |  | -0.24 (-0.35, -0.13) |  | -0.13 (-0.15, -0.11) |  | -0.06 (-0.07, -0.05) |  |
|  | Q1 | 2.32 (1.88, 2.77) |  | 0.56 (0.44, 0.69) |  | -0.20 (-0.31, -0.09) |  | -0.12 (-0.14, -0.10) |  | -0.06 (-0.07, -0.05) |  |
|  | Q1 vs. Q0 | 0.19 (-0.41, 0.78) | 0.53 | 0.02 (-0.15, 0.19) | 0.83 | 0.04 (-0.11, 0.19) | 0.58 | 0.01 (-0.02, 0.04) | 0.54 | 0.00 (-0.01, 0.01) | 0.96 |
|  | Q2 | 2.66 (2.21, 3.12) |  | 0.45 (0.32, 0.58) |  | -0.18 (-0.29, -0.07) |  | -0.11 (-0.13, -0.09) |  | -0.07 (-0.08, -0.06) |  |
|  | Q2 vs. Q0 | 0.53 (-0.08, 1.14) | 0.09 | -0.09 (-0.27, 0.08) | 0.29 | 0.07 (-0.09, 0.22) | 0.41 | 0.02 (-0.01, 0.05) | 0.17 | -0.01 (-0.02, 0.01) | 0.40 |
|  | Q3 | 2.89 (1.91, 3.87) |  | 0.61 (0.31, 0.91) |  | -0.35 (-0.62, -0.08) |  | -0.14 (-0.19, -0.09) |  | -0.05 (-0.08, -0.03) |  |
|  | Q3 vs. Q0 | 0.76 (-0.30, 1.82) | 0.16 | 0.07 (-0.26, 0.39) | 0.69 | -0.10 (-0.39, 0.19) | 0.49 | -0.01 (-0.06, 0.04) | 0.77 | 0.01 (-0.02, 0.03) | 0.64 |
| *LRRK2* | Q0 | 2.74 (1.74, 3.74) |  | 0.45 (0.17, 0.72) |  | 0.00 (-0.21, 0.21) |  | -0.17 (-0.21, -0.12) |  | -0.07 (-0.09, -0.05) |  |
|  | Q1 | 2.71 (1.79, 3.64) |  | 0.37 (0.10, 0.63) |  | -0.19 (-0.39, 0.00) |  | -0.12 (-0.16, -0.08) |  | -0.07 (-0.09, -0.05) |  |
|  | Q1 vs. Q0 | -0.03 (-1.32, 1.27) | 0.97 | -0.08 (-0.45, 0.29) | 0.68 | -0.19 (-0.47, 0.09) | 0.18 | 0.05 (-0.01, 0.11) | 0.12 | 0.00 (-0.03, 0.03) | 0.79 |
|  | Q2 | 2.04 (1.21, 2.88) |  | 0.32 (0.10, 0.63) |  | -0.09 (-0.26, 0.07) |  | -0.12 (-0.16, -0.08) |  | -0.07 (-0.09, -0.05) |  |
|  | Q2 vs. Q0 | -0.69 (-1.95, 0.56) | 0.27 | -0.13 (-0.47, 0.22) | 0.47 | -0.09 (-0.36, 0.17) | 0.48 | 0.05 (-0.01, 0.11) | 0.09 | 0.01 (-0.02, 0.04) | 0.64 |
|  | Q3 | 2.45 (1.48, 3.42) |  | 0.32 (0.07, 0.57) |  | -0.09 (-0.29, 0.10) |  | -0.14 (-0.19, -0.09) |  | -0.08 (-0.09, -0.05) |  |
|  | Q3 vs. Q0 | -0.29 (-1.63, 1.06) | 0.67 | -0.13 (-0.49, 0.24) | 0.49 | -0.09 (-0.38, 0.19) | 0.50 | 0.03 (-0.04, 0.09) | 0.39 | 0.00 (-0.03, 0.03) | 0.91 |
| *GBA* | Q0 | 2.96 (1.64, 4.29) |  | 0.48 (0.21, 0.75) |  | -0.31 (-0.54, -0.09) |  | -0.19 (-0.25, -0.15) |  | -0.08 (-0.09, -0.07) |  |
|  | Q1 | 2.01 (0.67, 3.34) |  | 0.62 (0.36, 0.88) |  | -0.25 (-0.47, -0.04) |  | -0.12 (-0.17, -0.07) |  | -0.07 (-0.09, -0.05) |  |
|  | Q1 vs. Q0 | -0.96 (-2.81, 0.90) | 0.30 | 0.14 (-0.23, 0.51) | 0.44 | 0.06 (-0.25, 0.37) | 0.69 | 0.07 (0.00, 0.15) | 0.04 | 0.01 (-0.01, 0.04) | 0.31 |
|  | Q2 | 2.84 (1.26, 4.42) |  | 0.39 (0.08, 0.71) |  | -0.24 (-0.51, 0.03) |  | -0.12 (-0.19, -0.06) |  | -0.06 (-0.09, -0.04) |  |
|  | Q2 vs. Q0 | -0.12 (-2.16, 1.91) | 0.90 | -0.09 (-0.49, 0.32) | 0.68 | 0.07 (-0.27, 0.42 | 0.68 | 0.07 (-0.01, 0.16) | 0.09 | 0.02 (-0.01, 0.05) | 0.16 |
|  | Q3 | 2.98 (0.02, 5.93) |  | 0.21 (-0.39, 0.80) |  | -0.09 (-0.59, 0.41) |  | -0.15 (-0.28, -0.02) |  | -0.10 (-0.15, -0.05) |  |
|  | Q3 vs. Q0 | 0.01 (-3.22, 3.25) | 0.99 | -0.27 (-0.93, 0.38) | 0.40 | 0.22 (-0.32, 0.77) | 0.41 | 0.05 (-0.09, 0.19) | 0.50 | -0.02 (-0.07, 0.03) | 0.45 |

**Supplemental Table 4: Change in MDS-UPDRS I and III, MoCA, and DAT-SPECT SBR for participants with sporadic, LRRK2, and GBA PD categorized by time to threshold quartile**

Slopes (unit per year) were estimated from linear mixed models. Fixed effects included time * (genetic form * SAA results, baseline measure for each corresponding marker, baseline age, years since original diagnosis to baseline, sex, race [two levels], ethnicity [two levels], and education [three levels]) and LEDD. Participant-level random intercepts and slopes with unstructured covariance, heteroscedastic by genetic form.

Abbreviations: PD, Parkinson's disease; TTT, time to threshold; LRRK2, leucine-rich repeat kinase 2; GBA, glucocerebrosidase; MDS-UPDRS, Movement Disorder Society Unified Parkinson's Disease Rating Scale; MoCA, Montreal Cognitive Assessment; DAT-SPECT, dopamine transporter imaging with single-photon emission computed tomography; SBR, specific binding ratio.

|  |  | **MDS-UPDRS III** |  | **MDS-UPDRS I** |  | **MoCA** |  | **Caudate SBR** |  | **Putamen SBR** |  |
| --- | --- | --- | --- | --- | --- | --- | --- | --- | --- | --- | --- |
| **Group** | **AUC Quartile** | **Slope/β (95% CI)** | **p-value** | **Slope/β (95% CI)** | **p-value** | **Slope/β (95% CI)** | **p-value** | **Slope/β (95% CI)** | **p-value** | **Slope/β (95% CI)** | **p-value** |
| Sporadic | Q0 | 2.43 (1.95, 2.91) |  | 0.41 (0.28, 0.55) |  | -0.16 (-0.28, -0.05) |  | -0.11 (-0.13, -0.09) |  | -0.06 (-0.08, -0.05) |  |
|  | Q1 | 2.36 (1.92, 2.79) |  | 0.55 (0.43, 0.67) |  | -0.16 (-0.26, -0.06) |  | -0.12 (-0.14, -0.10) |  | -0.06 (-0.07, -0.05) |  |
|  | Q1 vs. Q0 | -0.07 (-0.69, 0.55) | 0.82 | 0.13 (-0.04, 0.31) | 0.14 | 0.00 (-0.15, 0.15) | 0.97 | -0.01 (-0.04, 0.02) | 0.55 | 0.01 (-0.01, 0.02) | 0.49 |
|  | Q2 | 2.34 (1.90, 2.78) |  | 0.58 (0.45, 0.70) |  | -0.29 (-0.41, -0.19) |  | -0.14 (-0.16, -0.12) |  | -0.7 (-0.08, -0.05) |  |
|  | Q2 vs. Q0 | -0.09 (-0.72, 0.54) | 0.78 | 0.16 (-0.02, 0.35) | 0.08 | -0.13 (-0.29, 0.02) | 0.09 | -0.03 (-0.06, 0.00) | 0.05 | 0.00 (-0.02, 0.02) | 0.95 |
|  | Q3 | 2.90 (1.91, 3.89) |  | 0.62 (0.32, 0.92) |  | -0.34 (-0.61, -0.07) |  | -0.14 (-0.19, -0.09) |  | -0.06 (-0.08, -0.03) |  |
|  | Q3 vs. Q0 | 0.47 (-0.61, 1.56) | 0.39 | 0.21 (-0.12, 0.54) | 0.22 | -0.18 (-0.47, 0.11) | 0.23 | -0.03 (-0.08, 0.02) | 0.24 | 0.01 (-0.02, 0.04) | 0.46 |
| *LRRK2* | Q0 | 2.15 (1.33, 2.96) |  | 0.30 (0.09, 0.51) |  | -0.09 (-0.25, 0.07) |  | -0.12 (-0.16, -0.08) |  | -0.07 (-0.09, -0.05) |  |
|  | Q1 | 2.44 (1.48, 3.39) |  | 0.33 (0.05, 0.60) |  | -0.14 (-0.35, 0.07) |  | -0.13 (-0.17, -0.08) |  | -0.07 (-0.09, -0.04) |  |
|  | Q1 vs. Q0 | 0.29 (-0.92, 1.51) | 0.63 | 0.02 (-0.32, 0.36) | 0.89 | -0.05 (-0.31, 0.21) | 0.71 | -0.01 (-0.06, 0.05) | 0.82 | 0.00 (-0.03, 0.03) | 0.90 |
|  | Q2 | 3.01 (2.01, 4.01) |  | 0.53 (0.26, 0.81) |  | -0.09 (-0.30, 0.12) |  | -0.16 (-0.21, -0.11) |  | -0.08 (-0.09, -0.05) |  |
|  | Q2 vs. Q0 | 0.86 (-0.39, 2.11) | 0.17 | 0.23 (-0.10, 0.56) | 0.17 | 0.00 (-0.26, 0.26) | 0.99 | -0.04 (-0.09, 0.02) | 0.17 | -0.01 (-0.04, 0.02) | 0.67 |
|  | Q3 | 2.46 (1.49, 3.44) |  | 0.33 (0.08, 0.58) |  | -0.10 (-0.30, 0.10) |  | -0.14 (-0.19, -0.09) |  | -0.08 (-0.09, -0.05) |  |
|  | Q3 vs. Q0 | 0.32 (-0.91, 1.55) | 0.61 | 0.03 (-0.29, 0.34) | 0.87 | -0.01 (-0.26, 0.24) | 0.94 | -0.02 (-0.08, 0.03) | 0.41 | -0.01 (-0.04, 0.02) | 0.61 |
| *GBA* | Q0 | 2.42 (0.86, 3.99) |  | 0.45 (0.16, 0.74) |  | -0.31 (-0.56, -0.07) |  | -0.12 (-0.19, -0.06) |  | -0.06 (-0.09, -0.04) |  |
|  | Q1 | 2.21 (0.86, 3.57) |  | 0.56 (0.28, 0.84) |  | -0.21 (-0.44, 0.02) |  | -0.12 (-0.17, -0.06) |  | -0.07 (-0.09, -0.05) |  |
|  | Q1 vs. Q0 | -0.21 (-2.26, 1.84) | 0.84 | 0.11 (-0.29, 0.51) | 0.58 | 0.10 (-0.23, 0.43) | 0.54 | 0.01 (-0.07, 0.09) | 0.82 | -0.01 (-0.04, 0.02) | 0.53 |
|  | Q2 | 3.07 (1.73, 4.40) |  | 0.53 (0.26, 0.80) |  | -0.32 (-0.55, -0.09) |  | -0.20 (-0.25, -0.15) |  | -0.08 (-0.09, -0.06) |  |
|  | Q2 vs. Q0 | 0.64 (-1.39, 2.67) | 0.53 | 0.08 (-0.31, 0.47) | 0.67 | -0.01 (-0.34, 0.33) | 0.98 | -0.08 (-0.16, 0.00) | 0.06 | -0.02 (-0.04, 0.01) | 0.22 |
|  | Q3 | 2.96 (-0.03, 5.94) |  | 0.20 (-0.39, 0.81) |  | -0.09 (-0.59, 0.42) |  | -0.15 (-0.28, -0.02) |  | -0.10 (-0.15, -0.05) |  |
|  | Q3 vs. Q0 | 0.53 (-2.84, 3.91) | 0.75 | -0.24 (-0.91, 0.42) | 0.47 | 0.23 (-0.34, 0.79) | 0.42 | -0.03 (-0.17, 0.12) | 0.71 | -0.04 (-0.09, 0.02) | 0.15 |

**Supplemental Table 5: Change in MDS-UPDRS I and III, MoCA, and DAT-SPECT SBR for participants with sporadic, LRRK2, and GBA PD categorized by area under the curve quartile**

Slopes (unit per year) were estimated from linear mixed models. Fixed effects included time * (genetic form * SAA results, baseline measure for each corresponding marker, baseline age, years since original diagnosis to baseline, sex, race [two levels], ethnicity [two levels], and education [three levels]) and LEDD. Participant-level random intercepts and slopes with unstructured covariance, heteroscedastic by genetic form.

Abbreviations: PD, Parkinson's disease; AUC, area under the curve; LRRK2, leucine-rich repeat kinase 2; GBA, glucocerebrosidase; MDS-UPDRS, Movement Disorder Society Unified Parkinson's Disease Rating Scale; MoCA, Montreal Cognitive Assessment; DAT-SPECT, dopamine transporter imaging with single-photon emission computed tomography; SBR, specific binding ratio.

|  |  | **MDS-UPDRS III** |  | **MDS-UPDRS I** |  | **MoCA** |  | **Caudate SBR** |  | **Putamen SBR** |  |
| --- | --- | --- | --- | --- | --- | --- | --- | --- | --- | --- | --- |
| **Group** | **Fmax Quartile** | **Slope/β (95% CI)** | **p-value** | **Slope/β (95% CI)** | **p-value** | **Slope/β (95% CI)** | **p-value** | **Slope/β (95% CI)** | **p-value** | **Slope/β (95% CI)** | **p-value** |
| Sporadic | Q0 | 2.65 (2.20, 3.09) |  | 0.59 (0.46, 0.71) |  | -0.21 (-0.32, -0.10) |  | -0.13 (-0.15, -0.11) |  | -0.07 (-0.08, -0.06) |  |
|  | Q1 | 2.21 (1.76, 2.66) |  | 0.42 (0.29, 0.54) |  | -0.19 (-0.31, -0.09) |  | -0.11 (-0.13, -0.09) |  | -0.06 (-0.07, -0.05) |  |
|  | Q1 vs. Q0 | -0.44 (-1.04, 0.17) | 0.16 | -0.17 (-0.35, 0.00) | 0.06 | 0.02 (-0.14, 0.17) | 0.85 | 0.02 (-0.01, 0.05) | 0.18 | 0.01 (-0.01, 0.02) | 0.25 |
|  | Q2 | 2.26 (1.82, 2.69) |  | 0.55 (0.43, 0.68) |  | -0.21 (-0.32, -0.10) |  | -0.13 (-0.15, -0.11) |  | -0.06 (-0.07, -0.05) |  |
|  | Q2 vs. Q0 | -0.39 (-0.99, 0.21) | 0.20 | -0.04 (-0.21, 0.14) | 0.68 | 0.00 (-0.15, 0.16) | 0.96 | 0.00 (-0.03, 0.02) | 0.76 | 0.00 (-0.01, 0.02) | 0.64 |
|  | Q3 | 2.89 (1.91, 3.88) |  | 0.61 (0.31, 0.91) |  | -0.34 (-0.61, -0.07) |  | -0.14 (-0.19, -0.09) |  | -0.05 (-0.08, -0.03) |  |
|  | Q3 vs. Q0 | 0.25 (-0.82, 1.31) | 0.65 | 0.03 (-0.29, 0.35) | 0.88 | -0.13 (-0.42, 0.16) | 0.38 | -0.01 (-0.06, 0.04) | 0.64 | 0.01 (-0.01, 0.04) | 0.35 |
| *LRRK2* | Q0 | 2.44 (1.48, 3.39) |  | 0.27 (0.03, 0.51) |  | -0.04 (-0.23, 0.14) |  | -0.13 (-0.17, -0.09) |  | -0.07 (-0.09, -0.05) |  |
|  | Q1 | 2.29 (1.38, 3.19) |  | 0.41 (0.16, 0.65) |  | -0.15 (-0.34, 0.04) |  | -0.12 (-0.16, -0.07) |  | -0.07 (-0.09, -0.05) |  |
|  | Q1 vs. Q0 | -0.15 (-1.43, 1.13) | 0.82 | 0.14(-0.19, 0.47) | 0.41 | -0.11 (-0.37, 0.15) | 0.39 | 0.01 (-0.05, 0.07) | 0.68 | 0.00 (-0.03, 0.03) | 0.83 |
|  | Q2 | 2.53 (1.59, 3.47) |  | 0.46 (0.19, 0.72) |  | -0.12 (-0.32, 0.08) |  | -0.15 (-0.19, -0.10) |  | -0.07 (-0.09, -0.05) |  |
|  | Q2 vs. Q0 | 0.09 (-1.23, 1.41) | 0.89 | 0.19 (-0.17, 0.54) | 0.29 | -0.08 (-0.34, 0.19) | 0.57 | -0.02 (-0.07, 0.04) | 0.61 | 0.00 (-0.03, 0.03) | 0.79 |
|  | Q3 | 2.43 (1.44, 3.42) |  | 0.33 (0.08, 0.58) |  | -0.10 (-0.30, 0.09) |  | -0.14 (-0.19, -0.09) |  | -0.07 (-0.09, -0.05) |  |
|  | Q3 vs. Q0 | -0.01 (-1.35, 1.34) | 0.99 | 0.06 (-0.28, 0.39) | 0.74 | -0.06 (-0.33, 0.21) | 0.65 | -0.01 (-0.07, 0.05) | 0.71 | -0.01 (-0.04, 0.03) | 0.72 |
| *GBA* | Q0 | 1.40 (-0.04, 2.84) |  | 0.52 (0.21, 0.82) |  | -0.19 (-0.44, 0.05) |  | -0.11 (-0.17, -0.05) |  | -0.06 (-0.08, -0.04) |  |
|  | Q1 | 3.56 (2.19, 4.93) |  | 0.55 (0.27, 0.83) |  | -0.46 (-0.70, -0.21) |  | -0.19 (-0.26, -0.13) |  | -0.08 (-0.09, -0.05) |  |
|  | Q1 vs. Q0 | 2.16 (0.20, 4.12) | 0.03 | 0.03 (-0.38, 0.44) | 0.87 | -0.26 (-0.61, 0.08) | 0.13 | -0.08 (-0.17, 0.01) | 0.07 | -0.01 (-0.04, 0.01) | 0.35 |
|  | Q2 | 2.59 (1.39, 3.78) |  | 0.48 (0.21, 0.74) |  | -0.22 (-0.43, -0.01) |  | -0.16 (-0.21, -0.11) |  | -0.08 (-0.09, -0.06) |  |
|  | Q2 vs. Q0 | 1.18 (-0.67, 3.04) | 0.20 | -0.04 (-0.44, 0.36) | 0.84 | -0.02 (-0.35, 0.29) | 0.88 | -0.05 (-0.13, 0.03) | 0.19 | -0.02 (-0.04, 0.01) | 0.17 |
|  | Q3 | 2.95 (0.18, 5.72) |  | 0.20 (-0.40, 0.81) |  | -0.09 (-0.58, 0.41) |  | -0.15 (-0.29, -0.01) |  | -0.10 (-0.15, -0.05) |  |
|  | Q3 vs. Q0 | 1.55 (-1.58, 4.67) | 0.32 | -0.31 (-0.99, 0.36) | 0.46 | 0.11 (-0.45, 0.66) | 0.70 | -0.04 (-0.19, 0.11) | 0.58 | -0.04 (-0.09, 0.01) | 0.14 |

**Supplemental Table 6: Change in MDS-UPDRS I and III, MoCA, and DAT-SPECT SBR for participants with sporadic, LRRK2, and GBA PD categorized by fluorescence maximum quartile**

Slopes (unit per year) were estimated from linear mixed models. Fixed effects included time * (genetic form * SAA results, baseline measure for each corresponding marker, baseline age, years since original diagnosis to baseline, sex, race [two levels], ethnicity [two levels], and education [three levels]) and LEDD. Participant-level random intercepts and slopes with unstructured covariance, heteroscedastic by genetic form.

Abbreviations: PD, Parkinson's disease; Fmax, fluorescence maximum; LRRK2, leucine-rich repeat kinase 2; GBA, glucocerebrosidase; MDS-UPDRS, Movement Disorder Society Unified Parkinson's Disease Rating Scale; MoCA, Montreal Cognitive Assessment; DAT-SPECT, dopamine transporter imaging with single-photon emission computed tomography; SBR, specific binding ratio.
